# Spatiotemporal mapping of alloy mesostructure dynamics via multimodal coherent X-ray diffraction imaging

**DOI:** 10.1073/pnas.2513369122

**Published:** 2025-09-17

**Authors:** Shuntaro Takazawa, Kakeru Ninomiya, Minh-Quyet Ha, Tien-Sinh Vu, Yuhei Sasaki, Masaki Abe, Hideshi Uematsu, Naru Okawa, Nozomu Ishiguro, Kyosuke Ozaki, Takaki Hatsui, Taiki Hoshino, Maiko Nishibori, Hieu-Chi Dam, Yukio Takahashi

**Affiliations:** ^a^International Center for Synchrotron Radiation Innovation Smart, Tohoku University, Sendai, Miyagi 980-8572, Japan; ^b^Department of Metallurgy, Graduate School of Engineering, Tohoku University, Sendai, Miyagi 980-8579, Japan; ^c^RIKEN SPring-8 Center, Sayo-gun, Hyogo 679-5148, Japan; ^d^Institute of Multidisciplinary Research for Advanced Materials, Tohoku University, Sendai, Miyagi 980-8577, Japan; ^e^School of Knowledge Science, Japan Advanced Institute of Science and Technology, Nomi, Ishikawa 923-1211, Japan; ^f^Institute for Materials Research, Tohoku University, Sendai, Miyagi 980-8577, Japan

**Keywords:** coherent X-ray diffraction imaging, X-ray photon correlation spectroscopy, mesoscale dynamics, precipitation-strengthened alloy, magnesium alloy

## Abstract

Understanding mesoscale structural dynamics of precipitation-strengthened alloys is essential for optimizing the mechanical performances of these alloys. Herein, we establish a multimodal coherent X-ray diffraction imaging framework for spatiotemporal mapping of mesoscale structural dynamics in precipitation-strengthened alloys. As a demonstrative application, we visualized the structural evolution in Mg_97_Zn_1_Gd_2_ during isothermal annealing at 700 K, revealing real-time dynamics of nucleation, growth, and coarsening. Ptychographic reconstruction enabled imaging of microstructural transformations across a wide field of view (~100 μm^2^) with temporal resolution spanning several hours. We observed decomposition of (Mg, Zn)_3_Gd and concurrent precipitation and coarsening of long-period stacking ordered phases. To resolve local dynamics at finer spatiotemporal scales, we combined dynamic coherent diffraction imaging with X-ray photon correlation spectroscopy, targeting selected regions (~10 μm^2^) with time resolution down to tens of seconds. This approach revealed the rapid formation of nanoscale precipitates within 10 s after heating, followed by coarsening over several hundred seconds. Additionally, we applied optical flow analysis—a computational method to track motion patterns—to visualize and quantify the nucleation, growth, and coarsening kinetics. The abovementioned findings demonstrate the capability of in situ coherent X-ray techniques to acquire the real-time evolutions of mesoscale structures in complex materials. Our methodology offers a robust framework for investigating dynamic phenomena in diverse material systems, including metals, polymers, and functional nanomaterials, under realistic thermal or mechanical conditions.

## Significance

Understanding the mesoscale structural dynamics of precipitation-strengthened alloys is critically important for designing high-performance materials. In this study, we present a multimodal coherent X-ray diffraction imaging framework for spatiotemporal mapping of microstructural evolution during thermal processing. Using this method, we elucidate the nucleation, growth, and coarsening of nanoscale precipitates in a Mg-Zn-Gd alloy during isothermal annealing at 700 K. Our approach integrates ptychography, dynamic coherent diffraction imaging, and X-ray photon correlation spectroscopy to provide complementary insights across multiple spatial and temporal scales. Additionally, optical flow analysis enables quantitative characterization of transformation kinetics. Our methodology offers a robust framework for investigating dynamic phenomena in diverse material systems, including metals, polymers, and functional nanomaterials, under realistic thermal or mechanical conditions.

Precipitation-strengthened alloys are extensively utilized as structural materials in various fields, and formation, growth, and coarsening of nanoscale precipitates play crucial roles in enhancing the mechanical properties of these alloys (1). During the development of high-strength and high-durability materials from alloys for aerospace, automotive, and energy industries, controlling precipitation is essential for improving alloy performance (2). Precise understanding and regulation of the dynamics of microscopic precipitates are key to establishing design principles for optimized materials (3). For example, Mg alloys, known for their low densities and high specific strengths, are promising candidates for lightweight structural applications. However, conventional Mg alloys often suffer from inferior mechanical properties (4). To overcome these limitations, related research has focused on the addition of rare-earth and transition metal elements to Mg alloys to promote the precipitation of intermetallic compounds and production of long-period stacking ordered (LPSO) structures, thereby enhancing mechanical strength (5, 6). Mechanisms of formation of LPSO structures and the behaviors of nanoscale precipitates have been widely examined (7–11), and elucidating the evolutions of these structures is critical for advancing alloy design (12, 13).

Comprehensive understanding of these strengthening mechanisms requires direct visualization and quantitative analysis of precipitate generation, growth, coarsening, and spatial distribution in bulk alloys (14). Transmission electron microscopy (TEM) has been a powerful tool for observing nanoscale microstructures with high spatial resolution (15). Recently, 5-dimensional scanning transmission electron microscopy (5D STEM), which enables spatially and temporally resolved diffraction mapping, has emerged as a promising technique for analyzing nanoscale dynamic processes (16). However, both TEM and 5D STEM rely on thin-foil specimens, limiting their applicability to bulk materials. The small field of view and complex sample preparation can also introduce internal stresses or alter the intrinsic structure, complicating in situ analysis (17). Therefore, a more suitable measurement technique is needed to directly examine the dynamics of precipitates and evaluate global structural transformations in bulk alloys.

X-ray-based imaging techniques have been broadly employed to visualize mesoscale structural dynamics in bulk specimens. Due to their high penetration powers, X-rays are highly appropriate for investigating the internal structures of thick samples, which are often difficult to observe via TEM (18, 19). Transmission X-ray microscopy enables internal imaging based on X-ray absorption contrast; however, its applicability is limited for materials, such as Mg alloys, that exhibit low absorption contrasts. To address this limitation, coherent X-ray diffraction imaging (CXDI), which utilizes phase contrast, has garnered extensive attention (20–22). In CXDI, a coherent X-ray beam illuminates the sample, and diffraction patterns recorded by a two-dimensional detector are computationally reconstructed via phase retrieval algorithms to obtain the electron density distribution of the sample. This method facilitates nanoscale structure visualization with high spatial resolution, even in the cases of materials with low X-ray absorption contrasts. Among CXDI techniques, scanning CXDI—also termed X-ray ptychography—has been widely adopted in materials science owing to its ability to image large areas with exceptional spatial resolution (23, 24). Nevertheless, X-ray ptychography requires point-by-point scanning of the sample, which imposes limitations on temporal resolution. Consequently, alternative techniques that enable rapid structural observation are necessary for examining dynamic phenomena. Single-frame CXDI is one of these techniques, which offers superior temporal resolution despite its limited field of view as compared to that of ptychography (25–28). Moreover, time-constrained CXDI methods have recently been proposed in the X-ray regime, allowing dynamic structural analysis by incorporating temporal priors into phase retrieval (29–31).

X-ray photon correlation spectroscopy (XPCS) has been extensively employed to investigate dynamic fluctuations in materials (32, 33). By analyzing temporal fluctuations in the scattering patterns generated by coherent X-rays, XPCS provides statistical insights into structural dynamics over timescales ranging from submilliseconds to several hours (34–36). However, XPCS does not yield real-space images of the sample, rendering the resolution of the spatial distributions of dynamic structural changes using XPCS alone difficult. To overcome this limitation, we utilize single-frame CXDI to directly observe dynamic structural changes in real space. We refer to this approach as dynamic CXDI, which builds upon our previously reported method (28). We extended this technique by conducting high-temperature in situ dynamic CXDI experiments (37), enabling real-time observation of structural transformations in bulk alloys under thermal treatment conditions. Single-frame CXDI enables the reconstruction of the electron density distribution from a single diffraction pattern, thereby achieving high temporal resolution imaging. Furthermore, by applying XPCS to the diffraction patterns obtained through dynamic CXDI, we statistically characterize the internal dynamics of the sample (38, 39).

Here, we establish a multimodal X-ray imaging framework to directly visualize the spatiotemporal dynamics of nanoscale precipitates in a precipitation-strengthened Mg alloy. By integrating X-ray ptychography for wide-field structural mapping, in situ dynamic CXDI for real-time imaging, and XPCS for statistical dynamics analysis, we capture real-time transformations—from nucleation to coarsening—with 40 nm resolution and 10 s resolution. Furthermore, optical flow analysis reveals the motion patterns of individual precipitates, enabling quantitative mapping of mesoscale structural kinetics. This approach provides insights into how precipitates evolve under realistic thermal conditions, offering strategies for designing high-performance structural materials.

## Experimental Procedures.

**Fig. 1.**
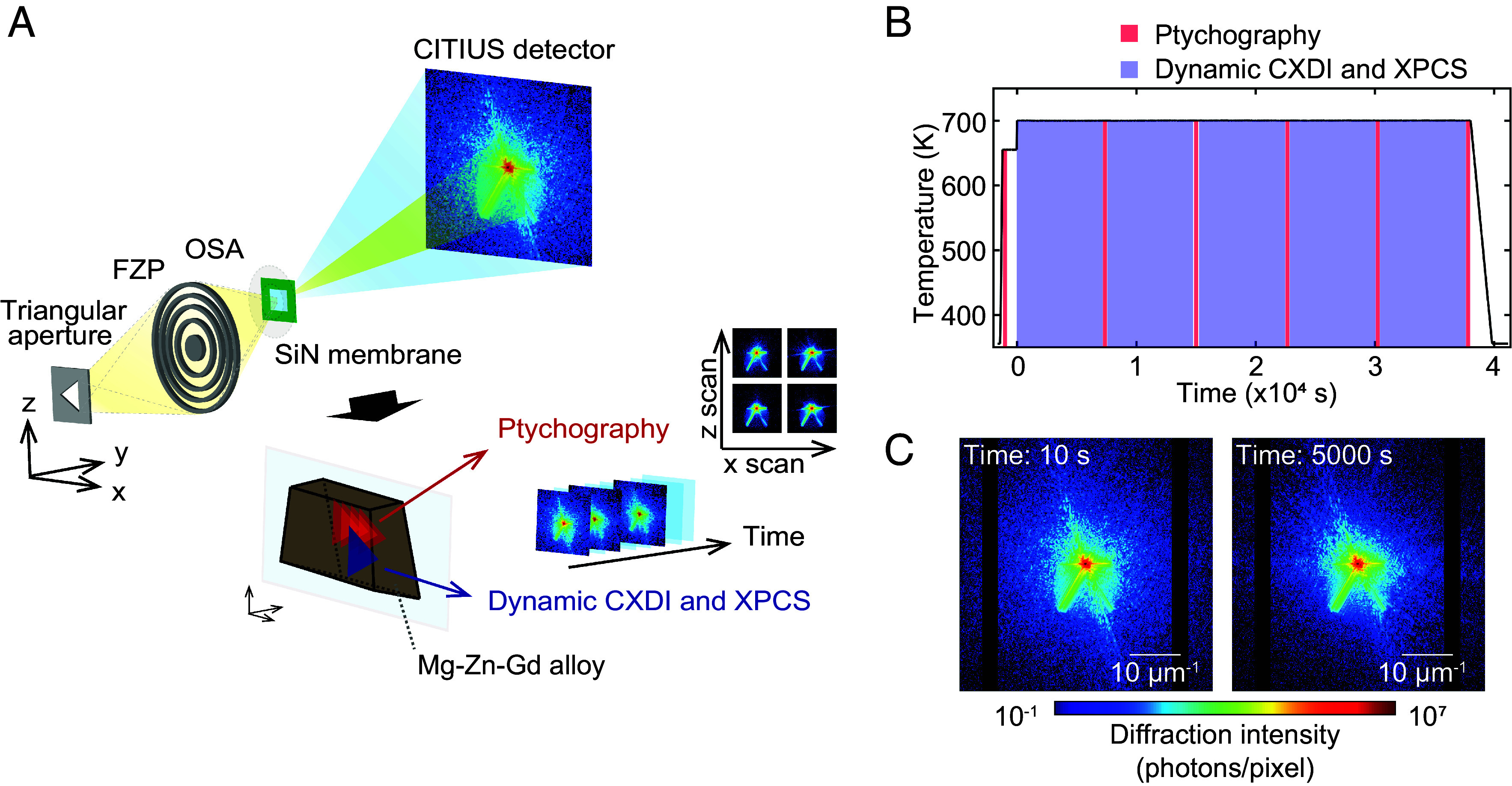
(*A*) Schematic of the experimental setup and data analysis workflow. (*B*) Heating profile of the sample. Time points indicated in blue represent data acquisition for XPCS and single-frame CXDI. Time points in red correspond to the data used for ptychographic reconstruction. (*C*) Representative diffraction patterns. Timestamps in the *Upper Left* corner of each pattern denote the elapsed time since the start of isothermal annealing at 700 K. Each pattern was obtained by summing 20 frames acquired with the CITIUS detector (effective exposure time: 0.5 s per frame), resulting in a total exposure time of 10 s per pattern.

Mg_97_Zn_1_Gd_2_ used in this study is categorized as a Type II LPSO Mg alloy, in which the LPSO phase forms via high-temperature annealing after casting (40). Following solution treatment at 793 K for 2 h, the microstructure of the alloy comprises α-Mg and (Mg, Zn)_3_Gd phases. Upon annealing at temperatures above 673 K, decomposition of (Mg, Zn)_3_Gd and subsequent LPSO phase formation occur (41). Coherent X-ray diffraction measurements were conducted at the undulator beamline BL29XU of the SPring-8 synchrotron radiation facility (42). As depicted in Fig. 1*A*, a 5-keV monochromatic X-ray beam was shaped using a triangular aperture (10 μm per side, 10 μm-thick Pt foil) and then focused by a Fresnel zone plate (FZP; diameter: 300 μm and outermost zone width: 50 nm, XRnanotech). FZP was positioned 100 μm above the aperture along the vertical axis, allowing only the +1st order diffracted beam to illuminate the sample. Undesired diffraction orders were blocked using an order-sorting aperture (OSA; 20 μm diameter, 20 μm-thick Pt foil) placed between FZP and the sample. The sample was illuminated with an X-ray beam having intensity distribution in the shape of a ~5 μm-sided triangle, with an incident flux of approximately 3 × 10^7^ photons/s. Diffraction patterns were recorded using a CITIUS detector (pixel size: 72.6 μm, 840 kpixels) located 3.3 m downstream of the sample (43), with an effective exposure time of 0.5 s per frame. Heating profile of the sample is shown in Fig. 1*B*. X-ray ptychography measurements were performed initially at room temperature, and subsequently at six times points corresponding to the red markers in Fig. 1*B*: immediately after heating to 655 K, and periodically during isothermal holding at 700 K. Scan parameters were 15 × 15 points, 500 nm step size, and 1 s exposure per point. In contrast, single-frame CXDI measurements were conducted at the time intervals indicated by pastel blue regions in Fig. 1*B*, corresponding to isothermal holding at 700 K. Herein, five sequential image acquisitions were performed, each lasting 2 h, and individual diffraction frames were obtained at 0.5 s exposure per frame. As depicted in Fig. 1*C*, the diffraction patterns evolved over time, indicating dynamic structural changes in the alloy microstructure.

## Results and Discussion

### High-Resolution Mesoscale Imaging by X-Ray Ptychography.

**Fig. 2.**
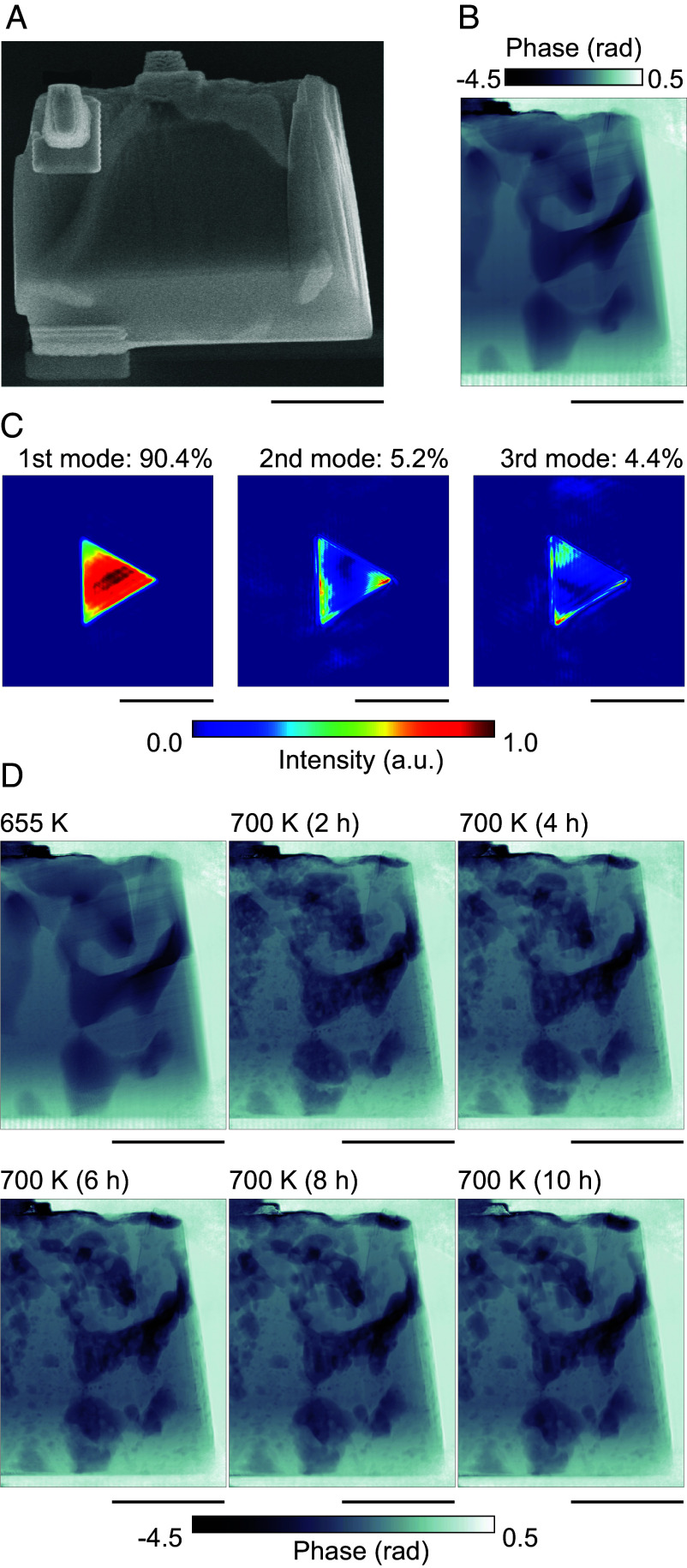
(*A*) SEM image of the sample. Trapezoidal features on the *Left* and *Upper* sides of the sample are fragments of the probe used to mount the specimen on the membrane. The rectangular region at the *Lower Left* represents W deposition used to fix the sample to the membrane. (*B*) Phase image of the sample before heating, obtained by ptychographic reconstruction. (*C*) Intensity distribution of the probe function obtained by ptychographic reconstruction. The population of each mode is indicated above each image. (*D*) Phase image of the sample during heating, also acquired via ptychography. Heating temperature and holding time are shown in the *Upper Left* part of each phase image. (Scale bars in all images (*A*–*D*) represent 5 μm.)

Fig. 2*A* shows a scanning electron microscopy (SEM) image of Mg_97_Zn_1_Gd_2_ before heating (JSM-IT510, JEOL). In the SEM image, the bright regions denote (Mg, Zn)_3_Gd, whereas the darker areas correspond to the α-Mg matrix. These phase distributions were confirmed by elemental mapping (*SI Appendix*, Fig. S1). Fig. 2 *B* and *C* show the reconstructed phase image and the intensity distribution of the probe function, respectively, obtained from the ptychographic measurements performed before heating. Three probe function images are shown in Fig. 2*C*, as mixed states were considered during the image reconstruction process (see *Materials and Methods* for details). The phase shift observed in the sample is approximately proportional to the projected electron density; therefore, larger phase shifts correspond to regions with higher electron density. Internal (Mg, Zn)_3_Gd structures, which were difficult to distinguish by SEM, were clearly visualized by CXDI with significantly high contrast and resolution. Fig. 2*D* shows the phase images obtained immediately after annealing Mg_97_Zn_1_Gd_2_ at 655 K and during subsequent isothermal holding at 700 K, recorded at intervals between 2 and 10 h. Spatial resolutions of the reconstructed images were estimated to be approximately 116.8 nm on average using phase retrieval transfer function (PRTF) (44). Comparative analysis of the ptychographic images revealed distinct microstructural evolution behaviors in the initial and advanced stages of annealing. Within the first 2 h after reaching a temperature of 700 K, rapid decomposition of (Mg, Zn)_3_Gd and formation of precipitates were observed, indicating fast transformation kinetics. This abrupt change has also been verified in separate heating experiments using another sample batch, indicating a similar conversion around 670 K (*SI Appendix*, Fig. S2). After the initial 2 h period, the decomposition of (Mg, Zn)_3_Gd more gradually proceeded and coarsening of the precipitates became the dominant feature of the structural evolution. In the field of view, sizes of the isolated precipitates in the α-Mg matrix reached sizes of approximately 0.5 to 1 μm. In the vicinity of (Mg, Zn)_3_Gd, numerous precipitates were observed, most probably corresponding to block-shaped LPSO phases. Previous studies have reported that (Mg, Zn)_3_Gd decomposes and transforms into LPSO structures over several tens of hours during annealing at 673 K, as detected by TEM (45). In this study, the elevated annealing temperature possibly accelerated (Mg, Zn)_3_Gd decomposition and precipitation in a few hours. Furthermore, precipitates were noticed in regions distant from (Mg, Zn)_3_Gd in the α-Mg matrix. Diffusion coefficients of Zn and Gd in Mg at 700 K are reported as $D_{Zn}$ = 4.09 × 10^−14^ m^2^/s and $D_{Gd}$ = 2.84 × 10^−15^ m^2^/s, respectively (46, 47), conforming to diffusion distances of approximately 34.3 μm for Zn and 9.0 μm for Gd over a 2-h period. Therefore, the structures observed in distant regions from (Mg, Zn)_3_Gd (Fig. 2*D*) are attributed to the precipitates that originated from not only the supersaturated α-Mg matrix due to the prior solution treatment but also Zn and Gd atoms released by the decomposition of (Mg, Zn)_3_Gd during annealing at 700 K.

### XPCS of Dynamic Structural Evolution.

**Fig. 3.**
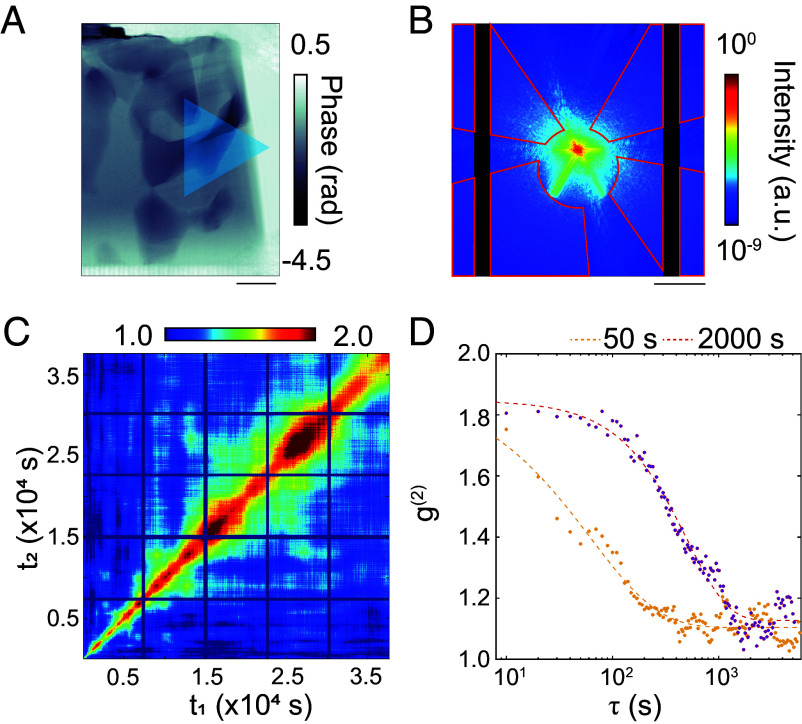
(*A*) Schematic showing the illuminated region on the sample. The phase image corresponds to the sample at room temperature before heating. (Scale bar, 2 μm.) (*B*) Region used for XPCS. This diffraction pattern was obtained by integrating the diffraction patterns continuously acquired over 10 h; the analyzed region is outlined in red. (Scale bar, 10 μm^−1^.) (*C*) Two-time correlation function (2-TCF) derived from diffraction data at a spatial frequency of 11.61 ± 0.18 μm^−1^. (*D*) Time correlation function (TCF) extracted from 2-TCF at a spatial frequency of 11.61 ± 0.18 μm^−1^, which is depicted in Fig. 3*C*. Legend indicates the elapsed time since the onset of annealing at 700 K.

**Fig. 4.**
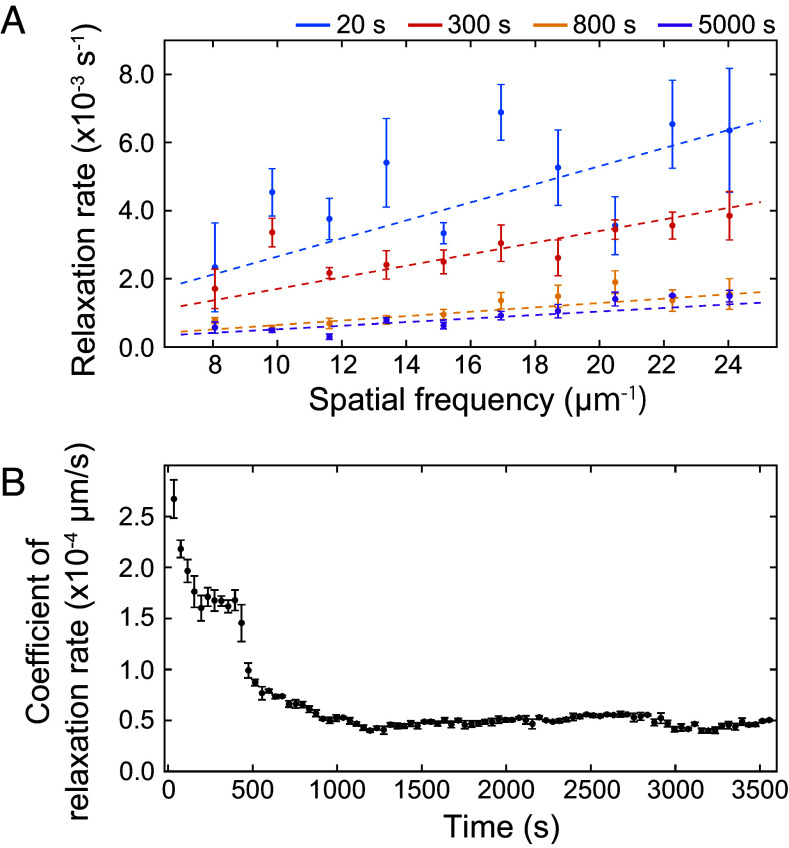
(*A*) Relaxation rate as a function of spatial frequency. Herein, 50 data points were obtained at intervals of 0.72 μm^−1^ and grouped into sets of 5. Solid circles denote the mean values, and error bars represent SE. Elapsed time since the start of isothermal annealing at 700 K is shown in the *Upper Right* part. (*B*) Time evolution of the dynamics speed parameter ***K***, representing the slope of the relaxation rate-spatial frequency relationship. For visibility, the data—originally calculated every 10 s—were averaged every 40 s. Solid circles indicate the average values, and error bars denote SD.

During the dynamic coherent X-ray diffraction intensity measurements, X-rays were continuously illuminated on the blue region shown in Fig. 3*A*, and a sequence of diffraction patterns was recorded. To conduct XPCS, these patterns were integrated to achieve an exposure time of 10 s, and the region enclosed by the red line in Fig. 3*B* was analyzed. The center position of the diffraction pattern for the XPCS analysis was set to the pixel with the maximum intensity, determined from a pattern recorded without the sample. Due to the off-axis configuration of FZP, the center of the circular projection formed by FZP is spatially offset from the zero-frequency position. The analysis region was carefully selected based on the signal-to-noise ratio and sensitivity to mesoscale dynamics. Specifically, the region within the circular projection from FZP was excluded due to insufficient diffraction intensity from the sample. Additionally, regions exhibiting strong scattering streaks originating from the sample contour and from the boundaries between the Mg matrix and large preexisting precipitates were also avoided. Fig. 3*C* shows two-time correlation function (2-TCF) calculated at the spatial frequency q = 11.61 ± 0.18 μm^−1^ using the following equation (48):

[1] $$C\left(t_{1},t_{2}\right)=\frac{{< <I\left(q,t_{1}\right)I\left(q,t_{2}\right)> >}_{\mathrm{pixel}}}{{< <I\left(q,t_{1}\right)> >}_{\mathrm{pixel}}< <I\left(q,t_{2}\right)> >{ª}_{\mathrm{pixel}}}.$$

where $I\left(q,t_{n}\right)$ represents the intensity of the diffraction pattern at q and the time $t_{n}$ and ${< <\cdot > >}_{\mathrm{pixel}}$ denotes an average over all pixels. In Fig. 3*C*, dark blue vertical and horizontal bands are visible, corresponding to missing data during ptychographic measurements. Careful examination of 2-TCF indicates that the relaxation time fluctuates instead of monotonically increasing with respect to the annealing duration. This demonstrates that the rates of decomposition of (Mg, Zn)_3_Gd and precipitation and growth of the LPSO phase in the illuminated region are temporally and spatially inhomogeneous. One-time correlation functions (TCF) were extracted from horizontal slices of the 2-TCF at distinct observation times using the conventional coordinate system approach (49). Fig. 3*D* depicts TCFs at 50 and 2,000 s after the onset of annealing, in which the time axis represents a delay time $\tau \left(s\right)=t_{1}-t_{2}$. TCF was fitted using the Kohlrausch–Williams–Watts (KWW) function:

[2] $$g^{\left(2\right)}\left(q,\tau \right)=\beta \left(q\right)\cdot \exp\left[-2\cdot {\left\{\Gamma \left(q\right)\cdot \tau \right\}}^{\alpha \left(q\right)}\right]+g_{\infty }(q),$$

where $\beta \left(q\right)$ is the contrast, $\Gamma \left(q\right)$ represents the relaxation rate (inverse of relaxation time), $\alpha \left(q\right)$ denotes the KWW exponent, and $g_{\infty }\left(q\right)$ is the baseline intensity. TCF analysis revealed that structural dynamics with relaxation times on the order of 100 s occurred within tens of seconds after annealing initiation. With the progress of annealing, these relaxation times increased. Spatial frequency and annealing time dependences of $\Gamma \left(q\right)$ were investigated in the q range from 7.35 to 24.74 μm^−1^ and annealing time range from 10 to 5,970 s using the KWW function. Fig. 4*A* shows the dependence of $\Gamma \left(q\right)$ on q at four representative annealing times, indicating an increase in $\Gamma \left(q\right)$ with an increase in q. By speculating a linear dependence of $\Gamma \left(q\right)$ on q, we modeled

[3] $$\Gamma \left(q\right)=Kq.$$

where K represents the dynamics “speed”. Annealing time dependence of K is depicted in Fig. 4*B*. K decreased over time, with a clear two-stage decrease centered around 400 s. After the second decrease, K stabilized around $0.5\times 10^{-4}$ μm/s. These results suggest that the dominant contributor to diffraction intensity changes transitions around 400 s, shifting from rapid decomposition and nucleation of (Mg, Zn)_3_Gd and LPSO phases to slower coarsening processes.

### Direct Observation of Dynamic Structural Evolution Using Dynamic CXDI.

**Fig. 5.**
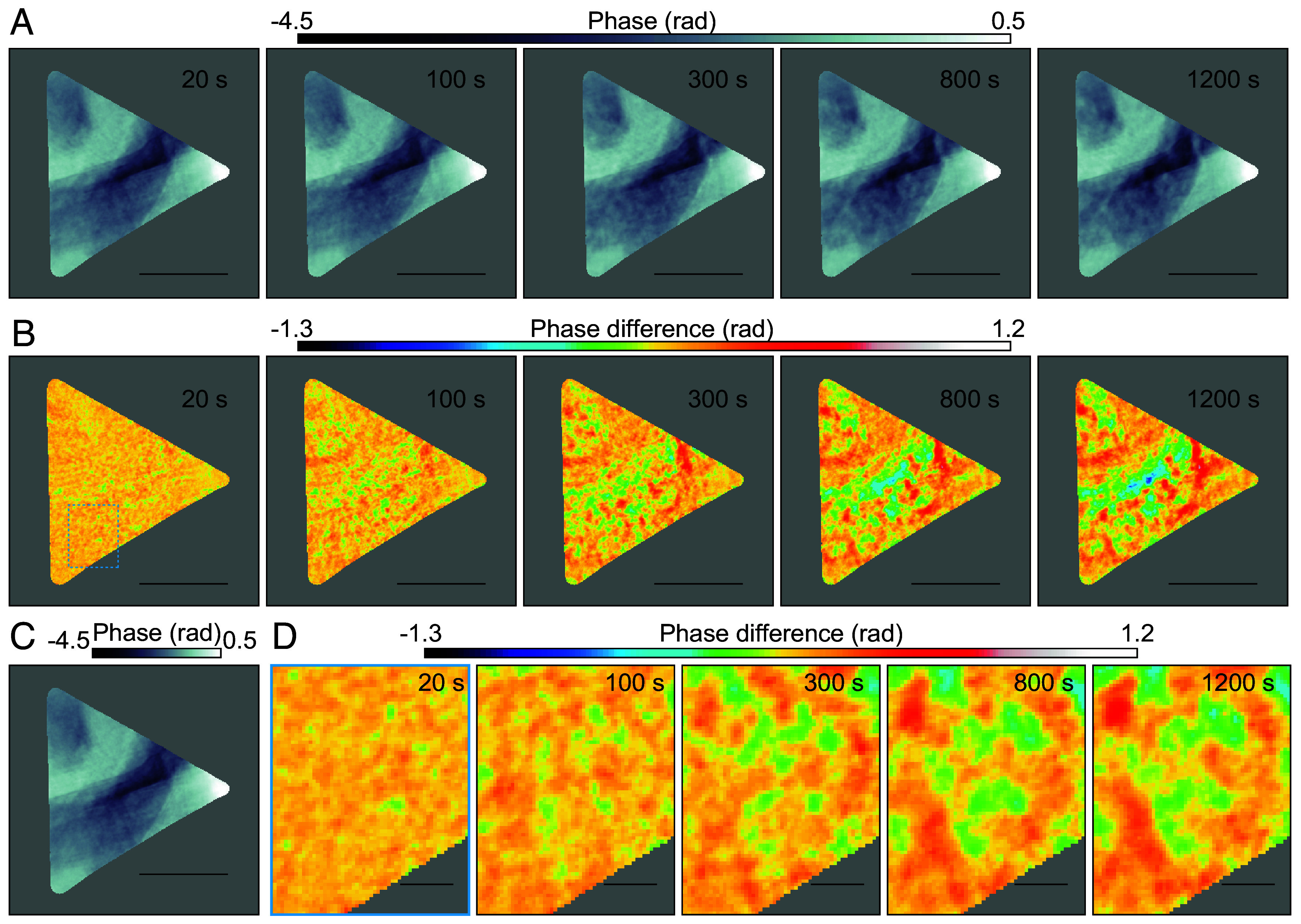
(*A*) Phase image reconstructed by single-frame CXDI. The illuminated region corresponds to the schematic depicted in Fig. 5*A*. (*B*) Difference image between two phase images. Image shown in Fig. 5*C* (acquired during heating from 655 to 700 K) was subtracted from that depicted in Fig. 5*A*. (*C*) Phase image used for evaluating the difference image in Fig. 5*B*. (*D*) Magnified view of the region outlined by blue dashed lines in Fig. 5*B*. (Scale bar, 2 μm for *A*–*C* and 300 nm for *D*.) Timestamps in the *Upper Right* part of each image (excluding Fig. 5*C*) represent the elapsed time since the onset of annealing at 700 K.

XPCS results demonstrated that unique dynamics occurred within the first 1,000 s of isothermal annealing. To directly examine microstructural changes during this period, we performed iterative phase retrieval reconstruction using the same integrated diffraction dataset as used in XPCS. Fig. 5*A* shows the reconstructed phase images at 20, 100, 300, 800, and 1,200 s after the initiation of annealing at 700 K. Sequences of the reconstructed phase images acquired from continuously recorded diffraction patterns are depicted in Movies S1–S5. Spatial resolutions of the reconstructed images, evaluated using PRTF, were estimated to be 42.1 nm on average. The spatial resolution achieved with single-frame CXDI was superior to that obtained with ptychography. This was due to the longer 10-s exposure time used in single-frame CXDI, which enabled photon detection at higher spatial frequencies, whereas ptychography employed a shorter 1-s exposure time per scan position. Comparison of these phase images revealed spatial changes in the phase-shift distributions within the dark regions corresponding to decomposing (Mg, Zn)_3_Gd. Difference images (Fig. 5*B*) were acquired by subtracting each phase image from the reference image (Fig. 5*C*). Fig. 5*D* depicts a magnified view of the blue dashed region in Fig. 5*B*. Intensities in the difference images imply variations in electron density. Green regions in Fig. 5*D* indicate high electron density, suggesting the production of precipitates, possibly the LPSO phase. Within the first 100 s after the onset of heating, nucleation and growth of small (~100 nm) precipitates were observed. After 300 s, overlapping of precipitates was detected, revealing coarsening. These results demonstrate that the decomposition of (Mg, Zn)_3_Gd and nucleation/growth of precipitates dominate in the initial stage (<300 s), whereas decomposition and coarsening dominate in the subsequent stages of heating.

**Fig. 6.**
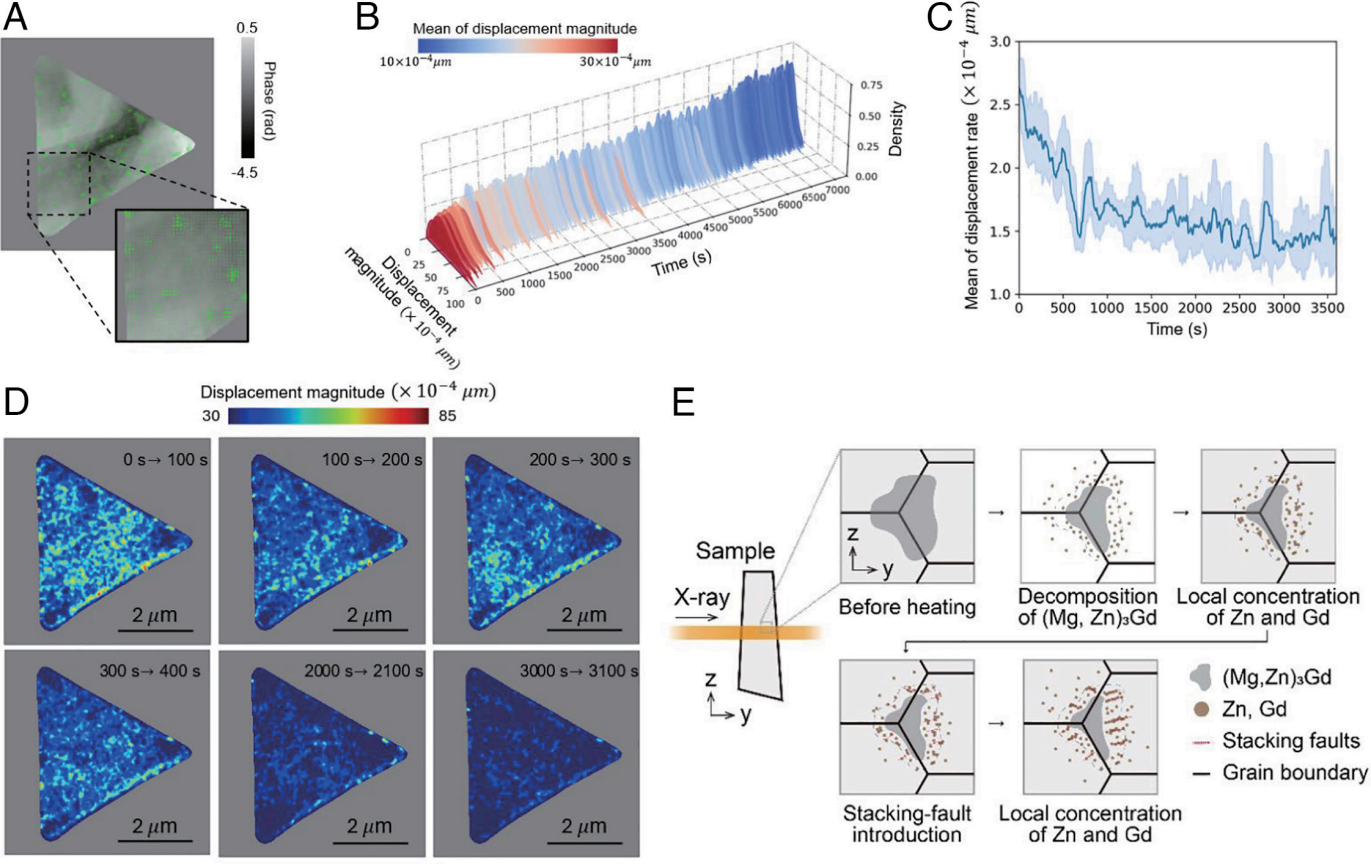
(*A*–*D*) Results of optical flow analysis applied to the reconstructed phase images: (*A*) Schematic of displacement vectors between timesteps of 10 and 20 s after annealing initiation at 700 K; (*B*) Probability density distribution of displacement magnitude at each holding time; (*C*) Average evolution rate based on the mean displacement magnitude over intervals of 10 s; and (*D*) Spatial and temporal mapping of microstructural evolution in the alloy, visualized as the sum of displacement magnitude at each pixel over the analyzed time periods (0 to 100, 100 to 200, 200 to 300, 300 to 400, 2,000 to 2,100, and 3,000 to 3,100 s), revealing where and when significant structural changes occur. (*E*) Schematic of the structural events occurring in the sample.

To obtain comprehensive insights into the timing and spatial locations of microstructural changes, we conducted optical flow analysis (50), a computational approach that quantifies local motion by tracking phase shift variations as vector fields, on dynamic CXDI video data, as detailed in *SI Appendix*, section S4. Fig. 6*A* shows an example of the displacement vector field extracted over the interval from 100 to 110 s after the initiation of annealing at 700 K; complete optical flow analysis covering the first 0 to 2 h after heating initiation is depicted in Movie S6. During the early stages, directional vectors clearly indicate the decomposition of (Mg,Zn)_3_Gd and successive formation of precipitates, revealing both the nucleation sites and timings of these processes. Fig. 6*B* shows the probability density distributions of displacement magnitudes calculated from optical flow vectors over consecutive 10-s intervals. These displacement magnitudes are in excellent agreement with theoretical diffusion distances evaluated from $D_{\mathrm{Gd}}$ (~107 nm), validating the reliabilities of the vector fields extracted by optical flow analysis. Fig. 6*C* depicts the temporal evolutions of average displacement rates during the first hour of annealing, indicating a continuous decrease in the rate of structural changes. This temporal behavior closely matches that of K independently achieved via XPCS (Fig. 4*B*). Fig. 6*D* shows a spatial-temporal map summarizing microstructural evolution, represented as cumulative displacement magnitudes per pixel over selected time intervals (0 to 100, 100 to 200, 200 to 300, 300 to 400, 2,000 to 2,100, and 3,000 to 3,100 s). During the initial 300 s, significant variations were observed near (Mg, Zn)_3_Gd, whereas no evident changes were noticed in α-Mg-dominated regions. This suggests that Zn and Gd diffusion is limited, leading to preferential precipitation near (Mg, Zn)_3_Gd. This behavior is consistent with high-temperature (723 to 773 K) TEM observations reported in previous studies (45). Based on the XPCS and dynamic CXDI results, we propose the mechanism of structural evolution during annealing at 700 K (Fig. 6*E*). Initially, rapid decomposition of (Mg, Zn)_3_Gd takes place, releasing Zn and Gd atoms into α-Mg. Due to high negative mixing enthalpy, Zn and Gd tend to locally aggregate (51). Decrease in K during the first ~400 s probably indicates this phase decomposition and localized enrichment. Furthermore, Zn and Gd reduce the stacking fault energy of α-Mg (52–54), facilitating stacking fault formation. These faults act as traps for diffusing Zn and Gd, resulting in further growth of enriched regions. This fault formation, growth, and eventual coarsening of enriched zones possibly correspond to the second decrease in K.

### Summary.

Herein, we investigated the microscopic structural changes occurring during isothermal annealing of precipitation-hardened Mg_97_Zn_1_Gd_2_ using high-temperature in situ coherent X-ray diffraction. X-ray ptychography clearly visualized the decomposition of (Mg, Zn)_3_Gd, precipitation of LPSO phases, and coarsening of precipitates during annealing at 700 K. Moreover, single-frame CXDI enabled systematic examination of the short-timescale production and growth of precipitates, whereas XPCS provided statistical insights into dynamic structural fluctuations according to time-resolved diffraction patterns. Optical flow analysis further quantified the growth rates and movement directions of precipitates, identifying the active regions of structural transformation.

Integration of these techniques not only deepens our understanding of precipitation strengthening mechanisms but also offers a powerful approach for real-time visualization and analysis of internal structural dynamics in materials. In the future, the extension of single-frame CXDI to three-dimensional (3D) tomography will facilitate more comprehensive investigation of the 3D morphologies and spatial distributions of precipitates, decomposition of (Mg, Zn)_3_Gd, and diffusion behaviors of Zn and Gd. Optical flow analysis is also applicable to 3D datasets, enabling the visualization of conversion velocities and directions as full 3D vector fields. In addition to single-frame CXDI, performing ptychographic tomography before and after in-situ heating can provide deeper insights. A major challenge in the present study for achieving 3D observation is the structural limitation of the sample holder with the heating system, which severely restricts the angular range of sample rotation and thereby impedes the acquisition of a dataset suitable for high-fidelity 3D reconstruction. Recent advances in gas-flow-type heating systems have enabled in situ nanotomography (55), but their integration into our vacuum-chamber-based setup remains challenging. Consequently, a critical next step toward enabling 3D imaging is the engineering of an advanced sample holder that accommodates large rotational angles. Given the availability of two-dimensional X-ray detectors which offer high frame rates and saturation count rates, the incident X-ray flux onto the sample is the primary factor limiting the spatiotemporal resolution of the present method. This spatiotemporal resolution can be significantly enhanced through the utilization of low-emittance synchrotron sources (56–58). Furthermore, the utilization of these sources will assist in 1) maintaining high spatiotemporal resolution during 3D tomography and 2) enabling structural analysis of LPSO phases via high-angle diffraction measurements. Although structural analysis of LPSO phases via high-angle diffraction measurements will necessitate the development of new multislice reconstruction algorithms (59, 60), the combined acquisition of low- and high-angle diffraction data is expected to yield insights into precipitate composition. This approach revealed the spatiotemporal progression of phase decomposition and LPSO formation in real time, offering fundamental insights into precipitation strengthening mechanisms. Looking ahead, extending this methodology to 3D tomography and applying it to a broader range of dynamic materials—including polymers, catalysts, and battery materials—will further advance our understanding of dynamic processes and enable the rational design of functional materials with tailored properties under operando conditions.

## Materials and Methods

### Sample Preparation.

Ingots of Mg_97_Zn_1_Gd_2_ were fabricated using a high-frequency induction heating system, followed by solution treatment at 793 K for 2 h. Following this treatment, a microsample with lateral dimensions of approximately 10 × 10 µm and a thickness of approximately 5 µm was extracted from the specimen using a focused ion beam (FIB, FB-2100, Hitachi). This microsample was subsequently mounted onto a Si_3_N_4_ membrane (HFN-1105, Norcada) designed for high-temperature in situ observation.

### Environmental Control.

Pure Mg sublimates at 703 K under a pressure of 1.07 Pa (61). To suppress sublimation while examining structural changes during heating up to 700 K, the sample chamber was initially evacuated to approximately 1 Pa and then backfilled with He gas to maintain a pressure at approximately 80 Pa.

### Radiation Dose.

Total dose in the Mg sample was calculated for the 10-h exposure with an incident flux of 3 × 10^7^ ph/s. Based on the linear absorption coefficient of Mg (2.74 × 10^2^ cm^−1^) (62), the density of Mg (1.738 × 10^3^ kg/m^3^) (63), and the volume of the irradiated region (approximately 54.1 µm^3^), the total dose was estimated to be approximately 1.3 × 10^9^ Gy. Subsequently, the resulting temperature increase was estimated under the assumption that the entire absorbed energy is converted to heat and distributed across both the Mg sample (approximately 13 × 10 × 5 µm) and the heating membrane with Si frame (approximately 5 × 5 × 200 µm). Using the specific heats of Mg (1.204 × 10^3^ J/kg/K) and Si (0.883 × 10^3^ J/kg/K) (64), and the density of Si (2.33 × 10^3^ kg/m^3^) (63), the temperature increase was estimated to be 0.012 K.

### Image Reconstruction.

For X-ray ptychographic reconstruction, we employed refractive ptychographic iterative engine (refPIE) (65), which considers mixed states (66), in combination with position correction algorithms (67–69). The same refPIE algorithm was also used for single-frame CXDI reconstructions, and no phase unwrapping was applied after phase retrieval.

Diffraction patterns were cropped to 560 × 560 pixels for reconstruction. Using the detector pixel size of 72.6 × 10^−6^ m, camera length of 3.3 m, and incident X-ray wavelength of 2.48 × 10^−10^ m, the pixel resolution at the sample plane was calculated to be 20.1 nm/pixel. For single-frame CXDI reconstruction, the illumination function was retrieved from prior ptychographic measurements and accordingly applied. Additionally, the sample image reconstructed from ptychography was utilized as the initial sample image for single-frame CXDI reconstruction.

## Supplementary Material

Appendix 01 (PDF)

Movie S1. Reconstructed phase images showing the initial evolution of microstructure in Mg_97_Zn_1_Gd_2_ during isothermal annealing at 700 K, from 10 s to 7160 s.

Movie S2. Phase image sequence from 7560 s to 14,740 s after the start of annealing at 700 K.

Movie S3. Phase image sequence from 15,270 s to 22,450 s at 700 K.

Movie S4. Phase image sequence from 22,850 s to 30,030 s at 700 K.

Movie S5. Phase image sequence from 30,420 s to 37,600 s at 700 K.

Movie S6. Time-resolved optical flow vector fields estimated between consecutive CXDI phase images during annealing of Mg_97_Zn_1_Gd_2_. Each frame displays the displacement vectors computed using the hybrid multi-scale optical flow algorithm, exhibiting the apparent motions of microstructural features. Flow fields highlight regions of dynamic activity, including precipitate evolution and local relaxation, with directional arrows representing the magnitude and orientation of displacement between adjacent time steps.

## Data Availability

All study data are included in the article and/or supporting information.
