# Supplementary material for: Spatiotemporal mapping of alloy mesostructure dynamics via multimodal coherent X-ray diffraction imaging
Source: Proc Natl Acad Sci U S A. 2025 Sep 17;122(38):e2513369122. doi: 10.1073/pnas.2513369122 (PMC12478095; doi:10.1073/pnas.2513369122)
Supplement: Supplementary file 1 — Appendix 01 (PDF) [file pnas.2513369122.sapp.pdf]

PNAS

# Supporting Information for
Spatiotemporal Mapping of Alloy Mesostructure Dynamics via Multimodal Coherent X-ray Diffraction Imaging

Shuntaro Takazawa, Kakeru Ninomiya, Minh-Quyet HA, Tien-Sinh VU, Yuhei Sasaki, Masaki Abe, Hideshi Uematsu, Naru Okawa, Nozomu Ishiguro, Kyosuke Ozaki, Takaki Hatsui, Taiki Hoshino, Maiko Nishibori, Hieu-Chi Dam, and Yukio Takahashi*

Yukio Takahashi
Email: ytakahashi@tohoku.ac.jp

**This PDF file includes**

Supporting text
Figures S1–S3
Legends for Movies S1–S6
SI References

**Other supporting materials for this manuscript are as follows:**

Movies S1–S6

## S1. Elemental mapping of $Mg_{97}Zn_1Gd_2$

Elemental mapping was performed using scanning electron microscopy coupled with energy-dispersive X-ray spectroscopy (SEM-EDS, JSM-IT510, JEOL) on the $Mg_{97}Zn_1Gd_2$ sample prior to the synchrotron X-ray radiation experiment. Secondary electron (SE) image (Fig. S1A) and corresponding SEM-EDS elemental maps indicate that the compounds are near the sample surface. Comparison of the SE image with the elemental maps reveals that the bright contrast regions in the SE image represent $(Mg, Zn)_3Gd$, whereas the dark contrast regions correspond to the α-Mg matrix.

## S2. Ptychographic observation of $Mg_{97}Zn_1Gd_2$ with stepwise temperature increase

Ptychographic analysis of a $Mg_{97}Zn_1Gd_2$ sample was conducted to investigate the temperature at which $(Mg, Zn)_3Gd$ undergoes significant decomposition. Note that the sample used for this observation is different from that described in the main manuscript and Fig. S1 despite being prepared using the same fabrication procedure. Optical configuration and X-ray energy are identical to those in the case of the experiment described in the main manuscript. Ptychographic measurements were performed at several temperatures during stepwise heating. Heating profile and SE image of the sample obtained before heating are shown in Fig. S2A and S2B, respectively. Figure S2C depicts the reconstructed phase images acquired by ptychographic reconstruction using the diffraction patterns obtained under different heating conditions. These phase images correspond to the region enclosed by the red dotted line in Fig. S2B. In these phase images, the darker contrast regions represent $(Mg, Zn)_3Gd$ due to its higher electron density as compared to that of the α-Mg matrix. Discrepancy in the contrast noticed between the SE and phase images suggests that some $(Mg, Zn)_3Gd$ species are located in the sample surface. Considerable decomposition of $(Mg, Zn)_3Gd$ was observed at temperatures exceeding 655 K. This indicated that the decomposition of $(Mg, Zn)_3Gd$ mentioned in the main manuscript initiated during the temperature ramp from 655 to 700 K.

## S3. Dynamic CXDI video data

Movies S1–S5 show the sequences of the reconstructed phase images achieved from continuously recorded diffraction patterns. Interval between data acquisition periods for each movie (e.g., between S1 and S2) is approximately 5 min. This gap represents the time needed for the ptychographic measurement. Timestamp depicted in the upper right corner of each movie is the elapsed time since the start of the continuous diffraction pattern collection. This starting point ($t = 0$) approximately coincides with the initiation of the temperature ramp from 655 to 700 K.

## S4. Optical flow analysis of the CXDI data of $Mg_{97}Zn_1Gd_2$

### Optical Flow

Optical flow analysis is a computational technique that estimates apparent motion between sequential image frames based on the brightness constancy assumption, which postulates that pixel intensity (or phase) values remain constant when the pixels move between frames [1]. Mathematically, $I(x, y, t)$ denotes the image intensity at the spatial coordinates $(x, y)$ at the time $t$, and the pixel moves by distance $(u, v)$ during the small time interval $\Delta_t$; this assumption can be expressed as follows:

$$I(x, y, t) \approx I(x + u, y + v, t + \Delta_t)$$

This equation states that the change in intensity over $\Delta_t$ at a point can be explained by a shift of the intensity pattern by $(u, v)$, which corresponds to physical motion of microstructural features. To determine the vector field $(u, v)$, we utilize the Farnebäck algorithm [2] on phase images. We emphasize that this analysis tracks apparent motions of phase patterns, which indicate real

displacements (for instance, movement of a precipitate and relaxation of strain) in the alloy microstructure.

**Hybrid Multi-Scale Optical Flow Strategy**

To accurately obtain both large-scale motions and fine-scale details, we implemented a hybrid multi-scale optical flow strategy using OpenCV's implementation of the Farnebäck algorithm. This algorithm finds the dense optical flow for all pixels in the frame via a polynomial expansion of local image neighborhoods. Specifically, this polynomial expansion involves fitting local intensity patterns around each pixel using polynomial functions, enabling accurate estimation of local displacements by analyzing shifts in these fitted patterns between consecutive frames. The method is controlled by numerous key hyperparameters including the averaging window size (*winsize*), which defines the neighborhood size for local polynomial fitting, and pyramid parameters (*pyr_scale* and *levels*), which establish a multi-scale hierarchy for resolving motion at different spatial resolutions.

Fundamental trade-off arises in the selection of hyperparameters for optical flow estimation, particularly when balancing the detection of larger-scale structural motion against the need to resolve small, precise displacements. Utilizing a large *winsize* in combination with a multi-level image pyramid enhances the robustness of algorithm to noise and enables the detection of broader, context-rich displacements by incorporating more spatial information. This helps to mitigate the aperture problem, where ambiguous local patterns, such as edges and low-texture regions, render the resolution of motion direction or magnitude difficult. However, this robustness is realized at the expense of spatial resolution, often leading to smoothing of small, localized motions. Contrarily, using a smaller *winsize* without pyramid layers better preserves fine-scale structural motion, it increases the sensitivity of the algorithm to noise and may result in unstable estimates in texture-poor regions, specifically near boundaries.

To integrate these competing effects, we employed a two-stage hybrid approach [3]. At first, we calculated an initial coarse optical flow field using a larger *winsize* (*20*) and pyramid structure (*pyr_scale = 0.5, levels = 3*) to firmly predict substantial motion patterns and reduce ambiguities, particularly at the borders of the triangular mask where local intensity patterns are limited. Thereafter, this coarse flow was used as an initial estimate for a second, fine-scale refinement step with a smaller *winsize* (*5*) utilizing the OPTFLOW_USE_INITIAL_FLOW flag in OpenCV. This refinement step improved the resolutions of subtle displacement features without the loss of the stability of the initial approximation.

To quantitatively validate the accuracy of this hybrid approach, we warped the original frame $I_t$ forward in time using the predicted displacement vectors to generate the estimated frame $\tilde{I}_{t+1}$, which was then compared with the actual subsequent frame $I_{t+1}$ using mean square error (MSE). Results demonstrated that the hybrid optical flow approach consistently reduced MSE by approximately 7% as compared to the cases of direct frame-to-frame differences (Fig. S3). As shown in Fig. S3, the hybrid method achieved a consistent reduction in MSE, averaging 7% improvement relative to those in the cases of direct frame-to-frame comparisons. This indicates that the hybrid multi-scale strategy effectively resolves the aperture problem while describing both coarse and fine motions, leading to more accurate and stable displacement fields, specifically near structurally complex regions including the triangular mask.

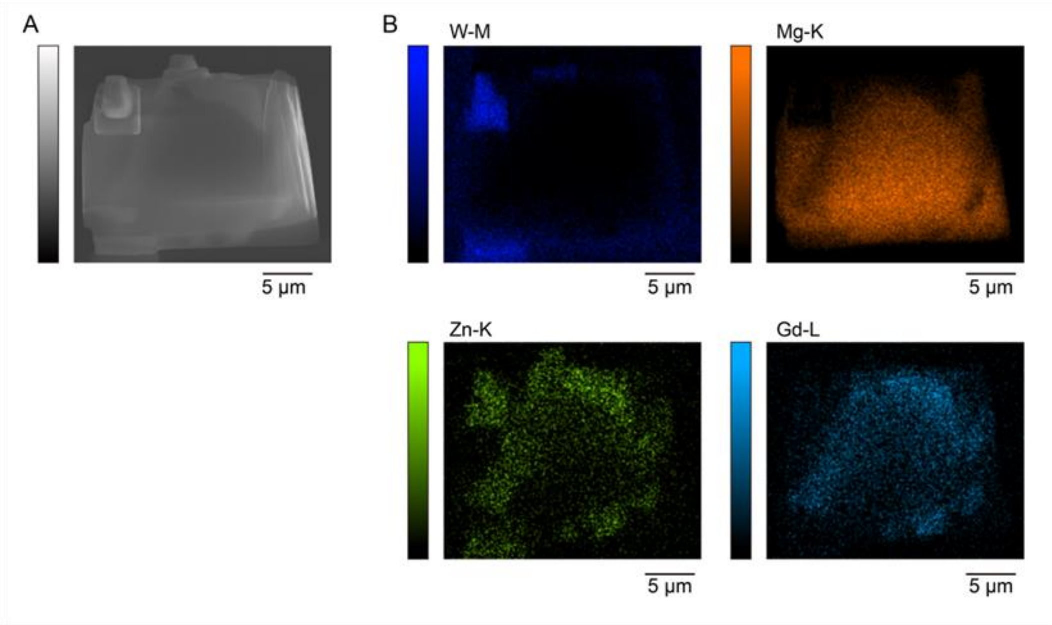


**Fig. S1.** SEM-EDS of the $Mg_{97}Zn_1Gd_2$ sample. (A) SE image. Although this image is nearly identical to Fig. 2A, it is included here to aid in visualizing the sample morphology, which can be difficult to discern from the elemental maps alone. (B) SEM-EDS elemental maps. Mapped element is indicated in the top left corner of each map. Both the SE image and elemental maps were acquired at an acceleration voltage of 25 kV.

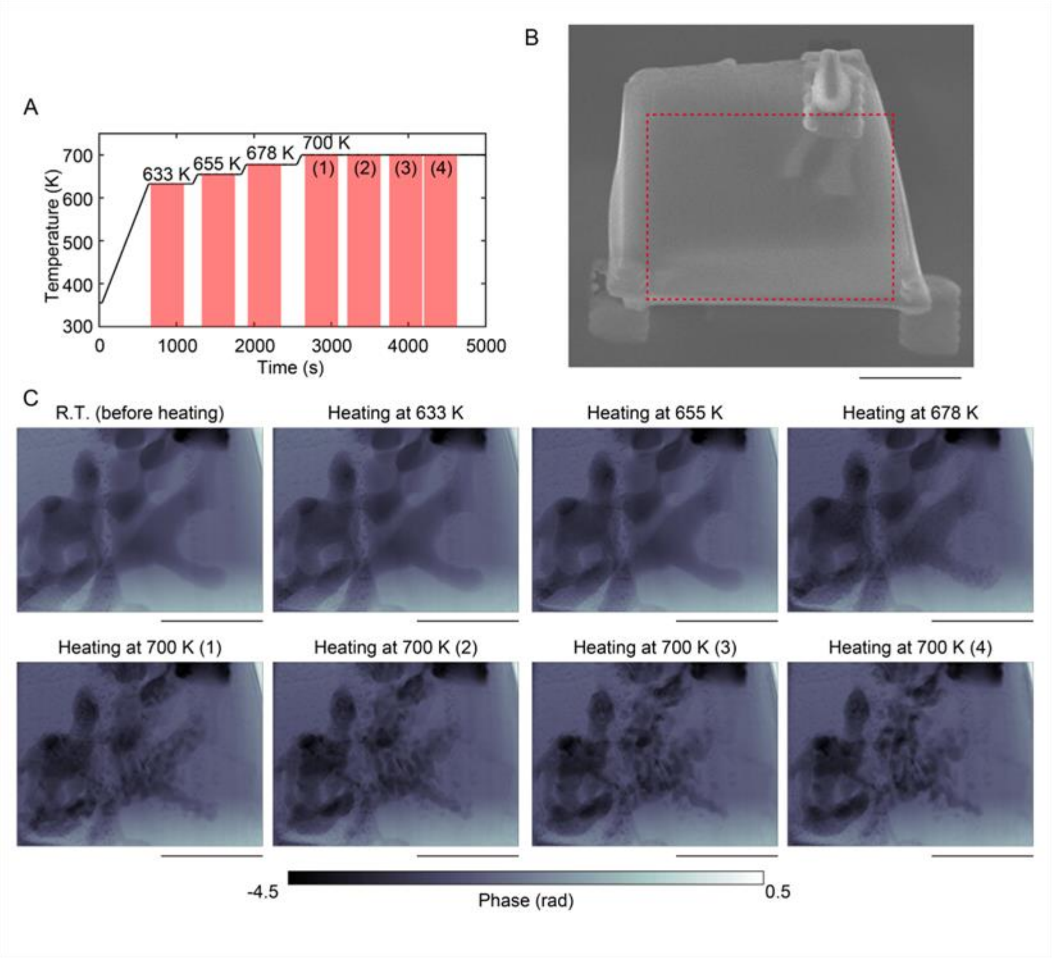


**Fig. S2.** (A) Heating profile for the sample described in section S2. Red-colored regions indicate the time intervals during which ptychographic measurements were performed. (B) SE image of the sample. (C) Reconstructed phase images. Temperature at the top of each image corresponds to the respective heating temperature marked on the profile in Fig. S2A. Scale bars for the SE and phase images are 5 µm.

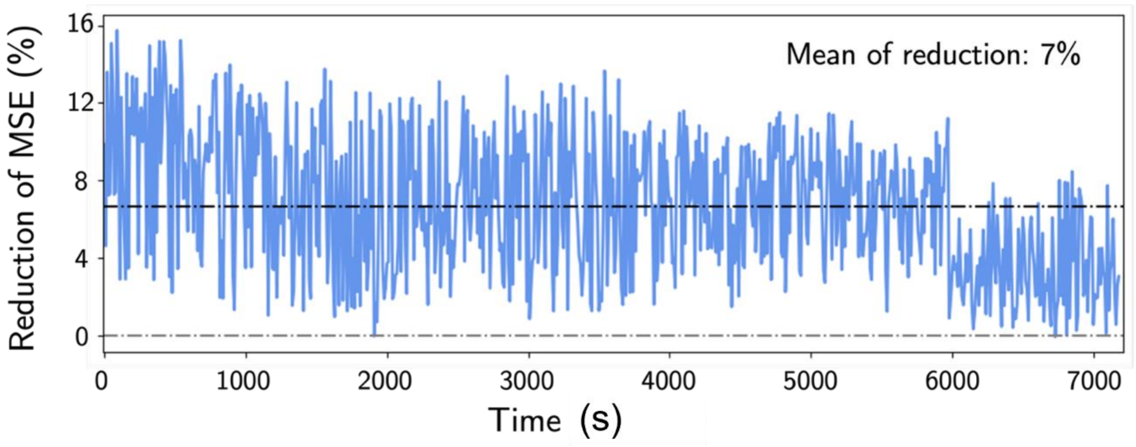


**Fig. S3.** Reduction in mean square error (MSE) achieved by the hybrid multi-scale optical flow strategy. Plot shows the difference between MSE computed directly from the original frame $\boldsymbol{I_t}$ and $\boldsymbol{I_{t+1}}$ and MSE evaluated between the predicted frame $\tilde{\boldsymbol{I}}_{\boldsymbol{t+1}}$ (obtained by warping $\boldsymbol{I_t}$ using optical flow) and $\boldsymbol{I_{t+1}}$. Positive values reveal a reduction in MSE due to flow-based warping. Black dashed line represents mean MSE reduction across all time steps.

## SI Appendix Movie Legends

**Movie S1 (separate file).** Reconstructed phase images showing the initial evolution of microstructure in $Mg_{97}Zn_1Gd_2$ during isothermal annealing at 700 K, from 10 s to 7160 s.

**Movie S2 (separate file).** Phase image sequence from 7560 s to 14,740 s after the start of annealing at 700 K.

**Movie S3 (separate file).** Phase image sequence from 15,270 s to 22,450 s at 700 K.

**Movie S4 (separate file).** Phase image sequence from 22,850 s to 30,030 s at 700 K.

**Movie S5 (separate file).** Phase image sequence from 30,420 s to 37,600 s at 700 K.

**Movie S6 (separate file).** Time-resolved optical flow vector fields estimated between consecutive CXDI phase images during annealing of $Mg_{97}Zn_1Gd_2$. Each frame displays the displacement vectors computed using the hybrid multi-scale optical flow algorithm, exhibiting the apparent motions of microstructural features. Flow fields highlight regions of dynamic activity, including precipitate evolution and local relaxation, with directional arrows representing the magnitude and orientation of displacement between adjacent time steps.

## SI References


1. B. K. Horn, B. G. Schunck, Determining optical flow. Artif. Intell. **17**, 185–203 (1981).
2. G. Farnebäck, Two-Frame Motion Estimation Based on Polynomial Expansion. In Proceedings of the 13th Scandinavian Conference on Image Analysis (SCIA)., 363–370 (2003).
3. D. Sun et al., Secrets of Optical Flow Estimation and Their Principles, IEEE. Conf. Comput. Vis. Pattern Recognit., 2432–2439 (2010).
